# Posterior Fusion With Pedicle Screw Cement Augmentation and Vertebroplasty Using Calcium Phosphate Cement for Osteoporotic Vertebral Fracture: A Case Report

**DOI:** 10.7759/cureus.75991

**Published:** 2024-12-19

**Authors:** Shunsuke Katsumi, Akira Shinohara, Daigo Arimura, Shintaro Obata, Mitsuru Saito

**Affiliations:** 1 Department of Orthopaedic Surgery, The Jikei University School of Medicine, Tokyo, JPN

**Keywords:** calcium phosphate cement, cement-augmented pedicle screws, minimally invasive spine stabilization, osteoporotic vertebral fracture, vertebroplasty

## Abstract

Osteoporotic vertebral fractures (OVFs) in elderly patients pose challenges due to bone destruction and surgical risks. This case report describes a minimally invasive approach using calcium phosphate cement (CPC) vertebroplasty and short fusion with cement augmentation of pedicle screws (CAPS) in a 91-year-old woman with severe OVF. The patient underwent CPC vertebroplasty at L1 and CAPS fixation at T12-L2, followed by osteoporosis medication. She regained mobility with no complications, screw loosening, or loss of correction at a two-year follow-up. We suggest this combined CPC-CAPS technique as a viable treatment option for OVF in high-risk elderly patients, providing minimal invasiveness and favorable long-term outcomes.

## Introduction

Osteoporotic vertebral fracture (OVF) is a pathology being treated increasingly frequently in Japan. Although various surgical techniques for OVF have been reported, problems such as screw loosening, loss of correction, and surgical invasiveness persist, and definitive solutions have yet to be established [1,2]. In this study, we report successful results from minimally invasive surgery using calcium phosphate cement (CPC) vertebroplasty and short fusion with cement augmentation of pedicle screws (CAPS) for OVF with severe bone destruction in patients over 90 years old.

## Case presentation

**Figure 1:**
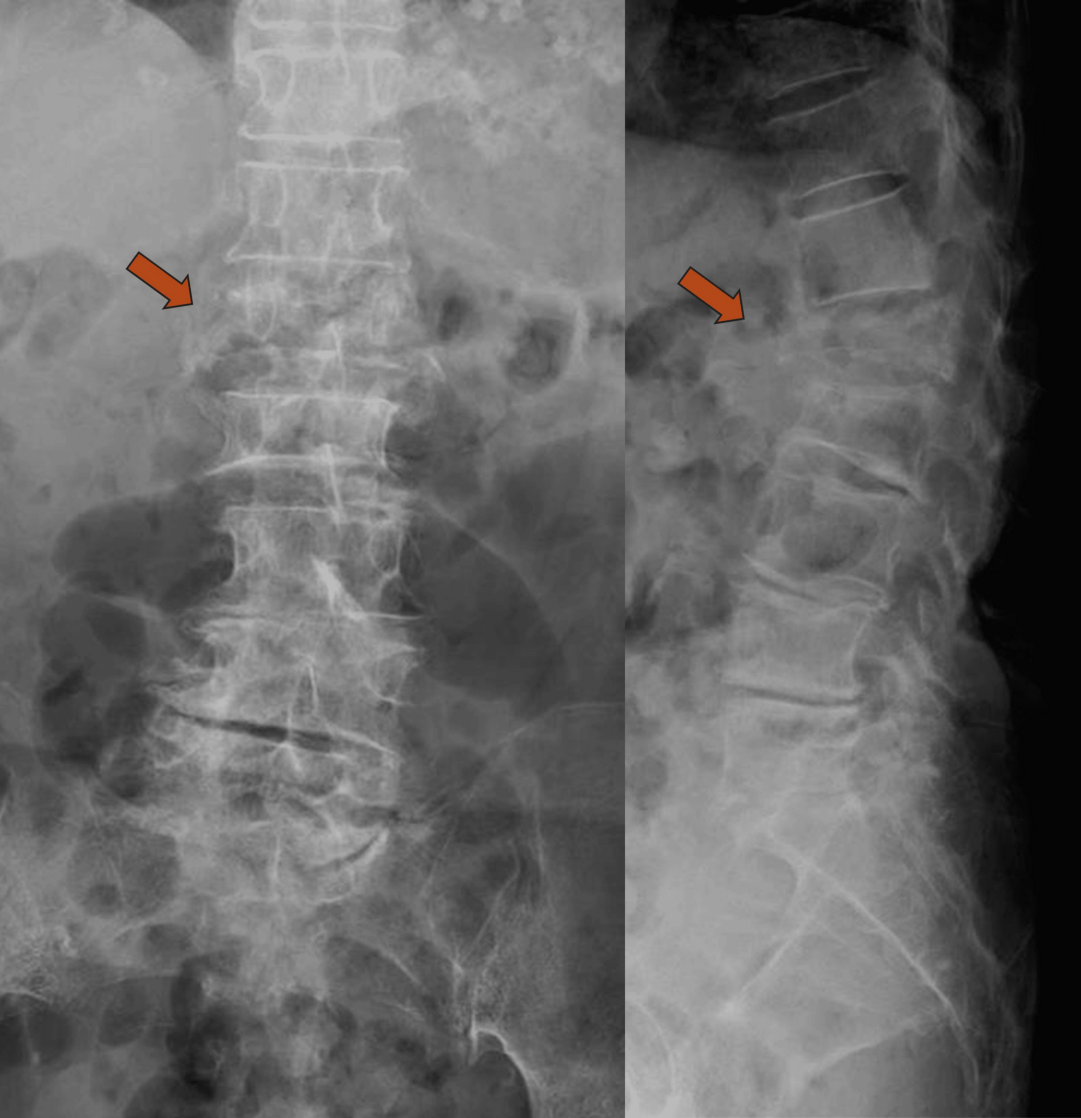
Preoperative X-ray showing an L1 compression fracture (arrow).

**Figure 2:**
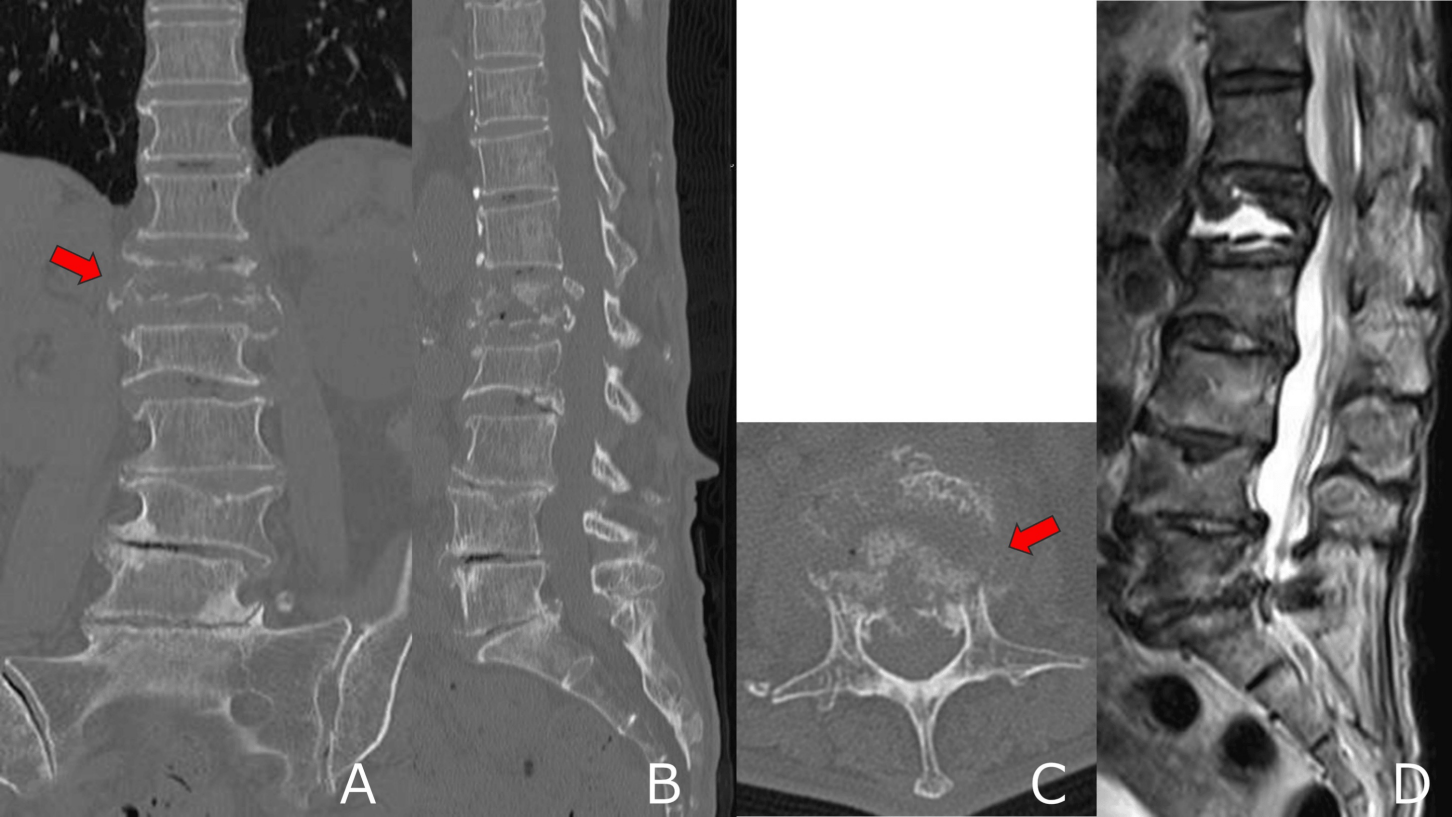
Preoperative computed tomography (CT) and magnetic resonance imaging (MRI). (A-C) CT images showing a vacuum cleft at L1. Bone cortical continuity of the vertebral body is not clear (arrow). (D) Sagittal T2-weighted MRI showing fluid retention in L1.

**Figure 3:**
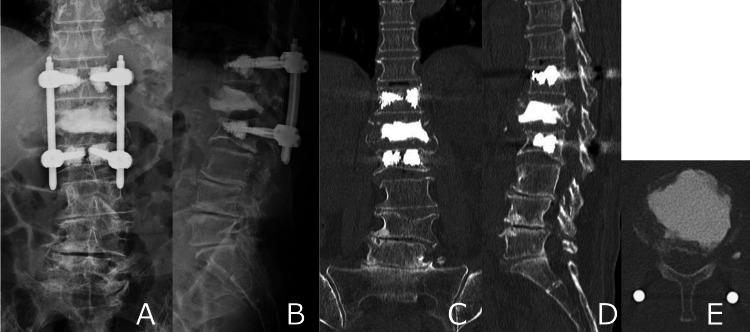
Postoperative imaging. (A–B) X-ray and (C–E) computed tomography (CT) images showing T12–L2 fixation with calcium phosphate cement and cement augmentation of pedicle screws.

**Figure 4:**
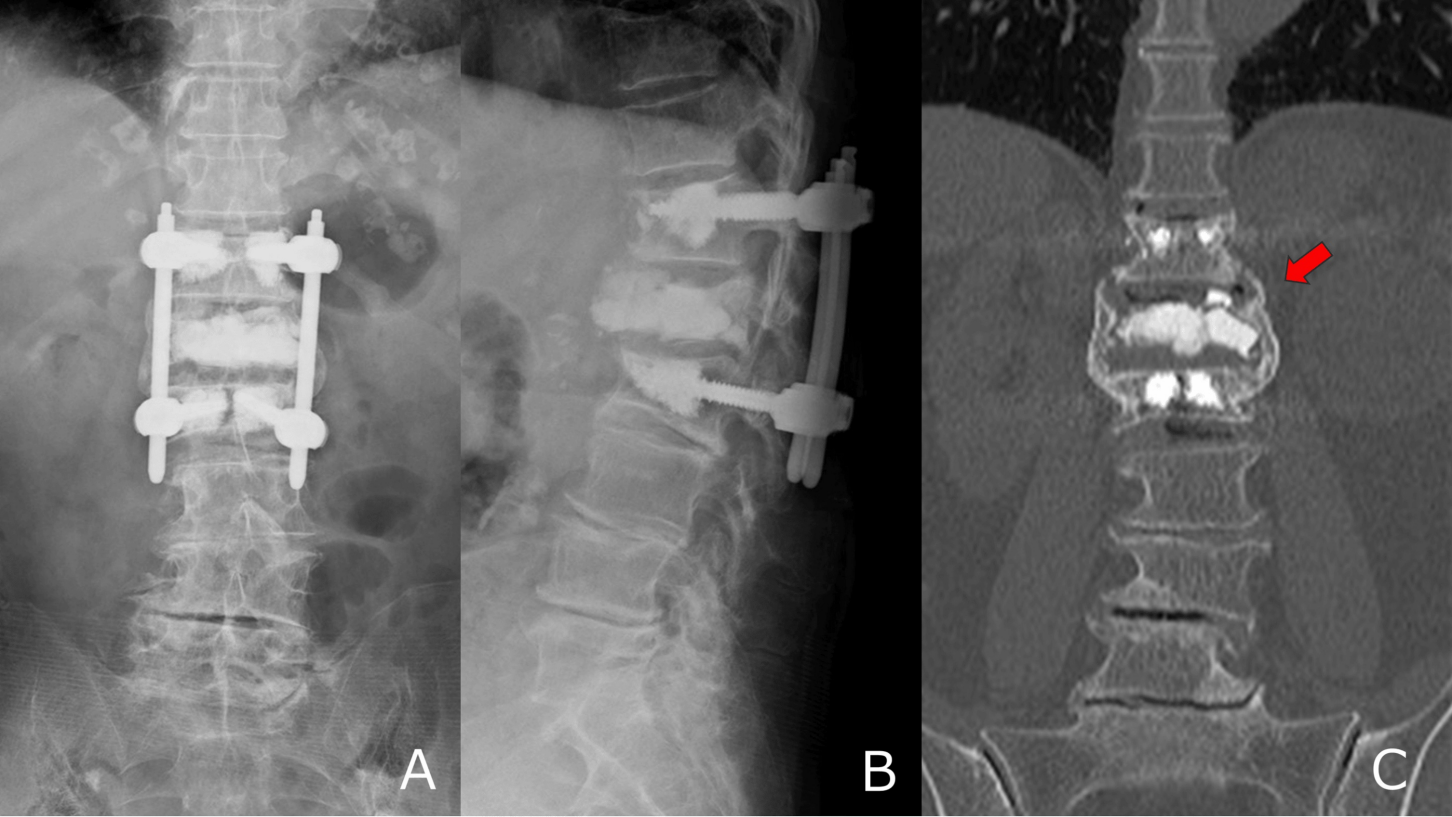
Postoperative imaging two years after surgery. (A–B) X-ray images showing no loss of correction. (C) Computed tomography (CT) image showing bony bridging on the lateral wall of the vertebral body (arrow).

A 91-year-old woman with hypertension and glaucoma was originally able to walk around her home. She became aware of unexplained back pain and visited her local physician, who diagnosed OVF at L1. The pain did not improve and gradually worsened, making walking difficult, and she was referred to our clinic. On physical examination, severe pain was observed in the lumbar region during body movement, but no motor or sensory disorders were detected. Bone density was 0.999 g/cm^2^ at L2-L4, but 0.739 g/cm^2^ at L1. X-ray showed a fracture at L1 (Figure 1), computed tomography (CT) showed a vacuum cleft in the vertebral body, and magnetic resonance imaging (MRI) showed a fluid sign (Figure 2). The patient underwent vertebroplasty using CPC (18 mL) at L1 and posterior fixation using CAPS (1 mL each) at T12 and L2 (Figure 3). The necrotic and scar tissues within the vertebral body were initially debrided and irrigated through the pedicle. To ensure the adequacy of debridement and confirm the absence of leakage beyond the vertebral body, contrast media was injected into the resulting cavity. After thoroughly aspirating blood and fluid, CPC was introduced into the cavity using a cement delivery gun. Subsequently, percutaneous pedicle screws were inserted into T12 and L2, followed by the injection of bone cement into the vertebral bodies through the screw tips. The total operative time was 1 hour and 49 minutes, with an estimated blood loss of 120 mL. Rehabilitation, including standing and gait training, was initiated on the day following surgery. The patient was able to walk from postoperative day 3 and was discharged on postoperative day 15. After surgery, romosozumab administration was initiated and continued for 12 months, followed by subsequent treatment with denosumab. Two years postoperatively, she could walk with no loosening of screws or loss of correction and bony bridging to the lateral side was seen due to bone fusion (Figure 4).

## Discussion

In 2007, Japan entered a super-aged society, characterized by a remarkable increase in the proportion of elderly individuals (aged 65 years or older) within the total population. By 2024, the proportion of elderly individuals reached a record high of 29.3%, the highest in the world [3]. This demographic shift indicates a continuing rise in age-related diseases, making the treatment of OVFs a critical concern for spinal surgeons. However, the lack of consensus on surgical strategies for OVFs poses challenges in decision-making. These challenges stem from a variety of factors, including patient demographics, comorbidities, fracture morphology, fixation range, and the selection of instrumentation. Moreover, elderly individuals over 90 years old often have multiple comorbidities, historically contributing to a reticence to perform surgery. This is because of the heightened risk of perioperative medical complications and implant failures [4]. This surgical procedure is a short fusion using the one above-one below technique, which has led to reduced surgical times and blood loss.

Although polymethylmethacrylate (PMMA) is often used in clinical practice, CPC has been reported to exhibit superior biocompatibility and osteogenic activity compared to PMMA [5]. CPC alters its composition to hydroxyapatite (HA), the primary inorganic component of bone, through a hydration reaction within the bone. HA possesses osteoconductive properties and has the characteristic of directly bonding with surrounding bone. Intravertebral curettage before vertebroplasty is important because the blood in the CPC reduces its compressive strength. In addition, PMMA is reported to have a higher propensity for cement leakage from the vertebral body compared to CPC [6], suggesting its favorable applicability in cases where continuity of the bone cortex on the anterior and lateral aspects of the vertebral body is lacking, as in this instance. In cases with severe vertebral destruction, the necessity of corpectomy has been reported; however, this procedure is highly invasive for elderly patients. This surgical procedure is anticipated to offer a less invasive, posterior-only approach for anterior column reconstruction.

However, the compressive strength of CPC is reportedly lower than that of PMMA [7], which is why combined screw usage is preferred in cases of severe bone destruction, such as in the present case. Therefore, in this case, a short fusion was performed using CAPS. Despite concerns regarding correction loss and screw loosening, CAPS has been reported to reduce the incidence of screw loosening by 78% [8]. Moreover, Yagi et al. reported increased pullout strength with CAPS, along with a reduction in implant-related reoperations [9]. Considering that the fracture site was at the thoracolumbar junction and the compressive strength of CPC is low, the use of cement augmentation in addition to pedicle screws may provide stronger stabilization. In this case, bone fusion was achieved, and no implant loosening was observed. This procedure may represent a viable treatment option for managing severe OVFs in the very elderly.

## Conclusions

For OVF in elderly patients with severe bone destruction, vertebroplasty with CPC and posterior fixation spanning one above-one below was performed with CAPS. The surgery was minimally invasive, resulted in no perioperative complications, achieved bone union, and maintained favorable outcomes. This technique could be one option for OVF treatment in an ultra-aging society.
